# Haemophagocytic syndrome in an adult suffering from pyrexia of unknown origin: an uncommon presentation of tuberculosis: a case report

**DOI:** 10.1186/s13104-017-2434-y

**Published:** 2017-02-27

**Authors:** Wasim Md. Mohosin Ul Haque, Md. Erfanur Rahman Shuvo, Muhammad Abdur Rahim, Palash Mitra, Tabassum Samad, Jalaluddin Ashraful Haque

**Affiliations:** 1 Department of Nephrology and Dialysis, Bangladesh Institute of Research and Rehabilitation in Diabetes, Endocrine and Metabolic Disorders (BIRDEM) General Hospital, Dhaka, 1000 Bangladesh; 2 Ibrahim Medical College, Shahbag, Dhaka, 1000 Bangladesh

**Keywords:** Case report, Haemophagocytic syndrome, Pyrexia of unknown origin, Tuberculosis

## Abstract

**Background:**

Tuberculosis is common, can involve various organs of the body and may have diverse presentations. Haemophagocytic syndrome is one of the rare presentations of tuberculosis carrying a very high mortality. Early detection and institution of anti-tuberculosis medications can be life-saving.

**Case presentation:**

A 23-year-old Bengali man presented with prolonged fever, weight loss, hepatosplenomegaly, pancytopenia and altered liver function. He had high erythrocyte sedimentation rate, positive tuberculin test, granuloma in liver biopsy, and haemophagocytosis was evidenced by histopathological examination of bone marrow. He recovered with anti-tuberculosis therapy.

**Conclusion:**

This case demonstrates that consideration of tuberculosis as an underlying cause of haemophagocytic syndrome could be rewarding and life-saving in this rapidly fatal condition.

## Background

Haemophagocytic syndrome (HPS) or haemophagocytic lymphangiohistiocytosis (HLH) is an uncommon disorder that may present with fever, lymphadenopathy and hepatosplenomegaly. Overactive macrophages phagocytose erythrocytes, leucocytes, platelets and their precursors in bone marrow and reticuloendothelial system. This syndrome can be primary or secondary. Primary HPS is a genetic disorder, occurs in younger age group. Secondary HPS may be triggered by viral infections like Epstein–Barr virus [1], but bacterial infections like tuberculosis (TB) is not uncommon [2–6]. Mortality ranges from 41 to 50% and in secondary HPS, delay in diagnosis increases mortality [7, 8]. Therefore, early recognition of the infective agent and treatment of the cause might be life-saving. We report this case to highlight TB as a cause of HPS in an adult patient and resolution of the disease with anti-TB treatment.

## Case presentation

A 23-year-old Bengali man presented with two-month history of intermittent fever, oral ulcer, anorexia and 9-kg weight loss. He gave history of several episodes of vomiting over five days before hospitalization. He had no other significant history of note, except frequent visit to kala-azar endemic area (Tangail) and sex with commercial sex worker two years back. He denied any history of contact with known TB cases. For his problems he consulted several physicians, underwent various investigations, took several courses of broad spectrum antibiotics and one course of anti-malarial drug without any benefit. His pre-admission investigation reports were insignificant except raised alanine aminotransferase (ALT) (142 U/L) and ultrasonographic evidence of hepato-splenomegaly.

The patient was very ill [World Health Organization (WHO) performance status—Grade 3] and wasted with a body mass index (BMI) of 16.7 kg/m^2^, febrile (temp of 102 °F), pulse 112/min, blood pressure 130/80 mm Hg. He had an oral ulcer (1 cm × 1 cm) on the inner side of left cheek with regular margin and whitish surface without any local lymphadenopathy. He had 7-cm smooth-surfaced, firm, tender hepatomegaly and 3-cm splenomegaly without ascites. Other examination findings including chest, precordium and ocular fundi were normal.

**Fig. 1:**
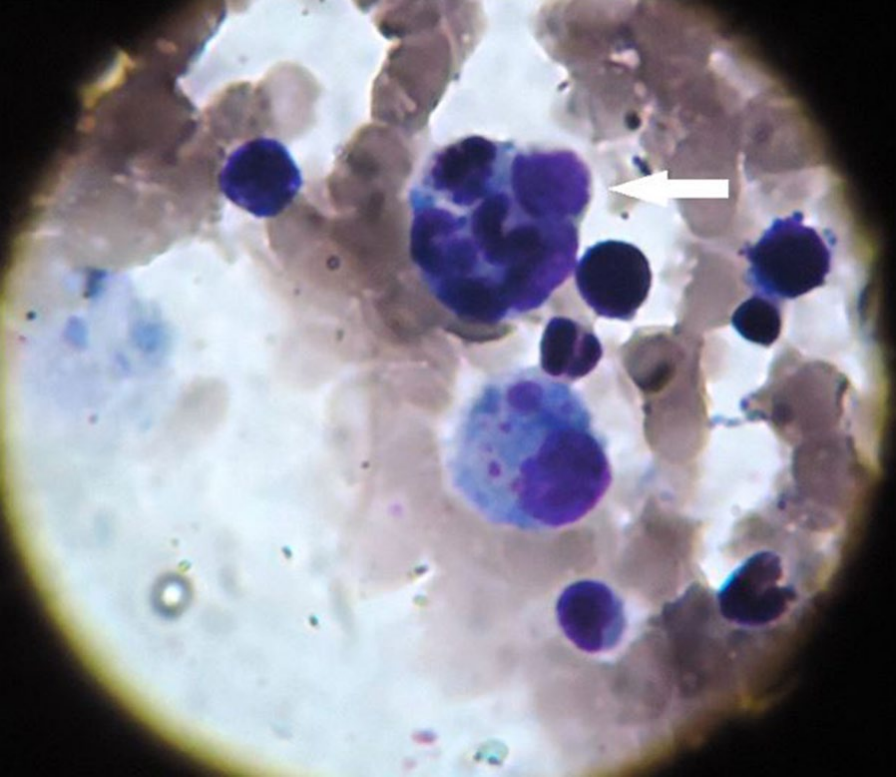
Bone marrow showing large histiocytes containing multiple concave nuclei of engulfed myeloid series cells (*white arrow*) suggestive of haemophagocytic syndrome

**Fig. 2:**
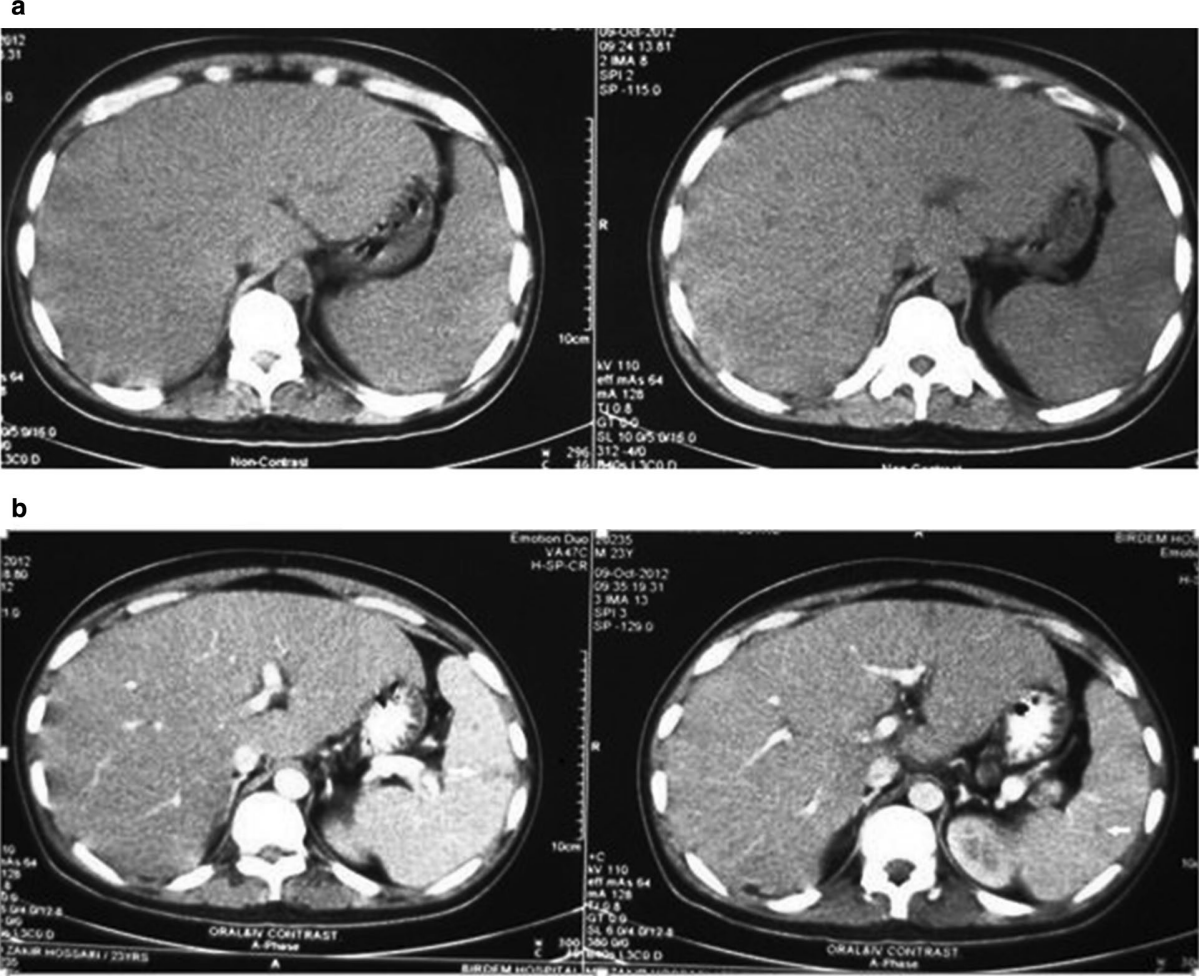
**a**, **b** Computed tomographic (CT) scan of abdomen showing hepatosplenomegaly with multiple isodense lesions in liver and spleen

**Fig. 3:**
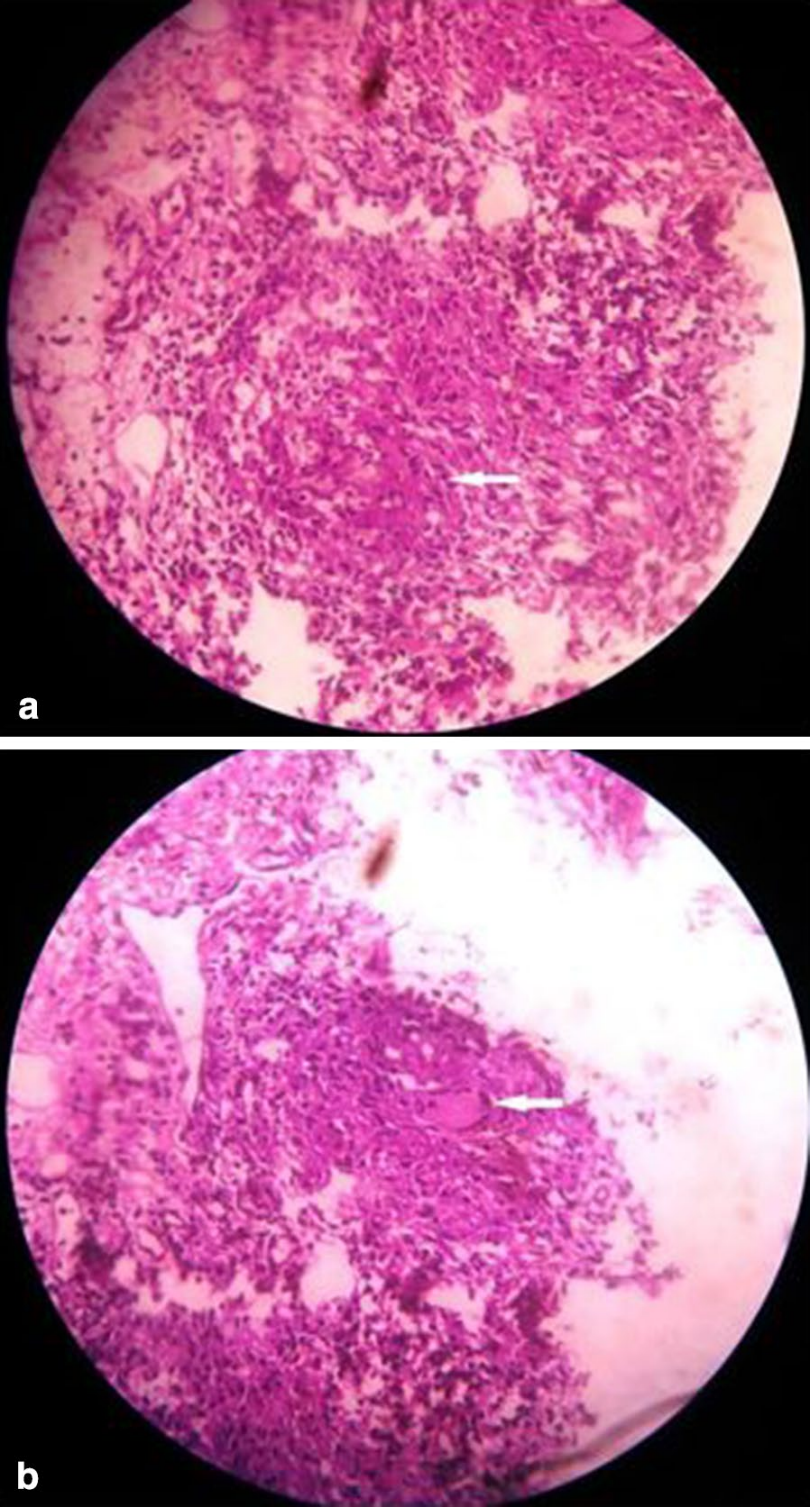
**a**, **b** Liver biopsy showing non-caseating granuloma with Langhan’s giant cell (*white arrow*) consistent with tuberculosis

His haemoglobin was 10.3 gm/dL, normochromic-normocytic and erythrocyte sedimentation rate (ESR) was 150 mm in 1st hour. Hepatic enzymes were raised (ALT 120 U/L, aspartate aminotransferase (AST) 132 U/L, alkaline phosphatase 982 U/L, gamma-glutamyl transferase (γ-GT) 1097 U/L, bilirubin 1.1 mg/dL) as were lactate dehydrogenase (LDH) (1286 U/L) and serum ferritin (3237 ng/mL). Abdominal ultrasonography revealed hepatosplenomegaly. Immunochromatography (ICT) for kala-azar was negative as were antibody against human immunodeficiency virus [anti-HIV (1 + 2)]. Tuberculin test revealed 15 mm induration at 72 h. Bone marrow examination revealed large histiocytes containing multiple concave nuclei of engulfed myeloid series cells (Fig. 1) suggestive of haemophagocytic syndrome. Biopsy from oral ulcer did not reveal any granuloma or malignancy. Viral serology e.g. IgG for CMV was positive but IgM was negative as was Monospot test. Computed tomography (CT) of abdomen revealed hepatosplenomegaly with multiple isodense lesions in liver and spleen (Fig. 2). Liver biopsy revealed non-caseating granuloma with Langhan’s giant cell consistent with tuberculosis (Fig. 3). He had raised triglyceride levels (2.64 mmol/L).

**Fig. 4:**
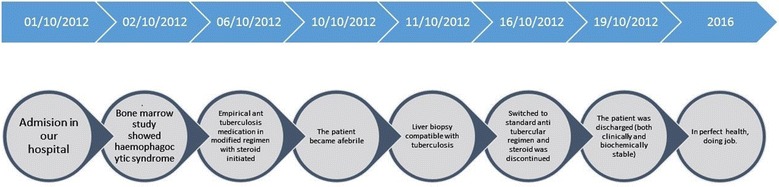
Timeline

So, the patient was finally diagnosed as having HPS due to disseminated TB. As the patient was seriously ill and had raised ALT, modified regime of anti-TB chemotherapy (including isoniazide, ethambutol, streptomycin and levofloxacin) along with prednisolone 40 mg/day was started. He became afebrile on 3rd day of starting anti-TB drugs but his haematological parameters deteriorated (Hb 8.8 gm/dL, total white cell counts 2420/cmm) requiring 2 units of blood transfusion. On 2nd week of anti-TB therapy, his clinical condition, haematological and biochemical parameters improved (Hb 12.1 gm/dL, total white cell counts 5430/cmm, ALT 87 U/L, AST 80 U/L, alkaline phosphatase 540 U/L) and we could switch him to standard anti-TB drugs and prednisolone was discontinued. He was discharged on 4th day of standard anti-TB medication. The patient received 6-months anti-TB medications uneventfully and completely cured (Fig. 4).

## Discussion

Primary HPS occurs in young patients and usually associated with genetic disorders [9, 10]. Secondary HPS affects people of any age. The causes may be variable ranging from infections through autoimmune disorders to malignancy [3]. Overall 3% of all HPS cases are associated with TB [11] and Tseng et al. [12] found that one-fourth of infection associated HPS among Taiwanese were due to *Mycobacterium tuberculosis*.

Presenting features of HPS mimics infection, liver disease, haematological malignancies or even encephalitis. A Swedish study reported fever to be the most prominent early feature, followed by hepatomegaly, splenomegaly, neurologic symptoms, rashes and lymphadenopathy in primary HPS [9]. Laboratory parameters include cytopenias, increased ferritin level, morphological evidence of haemophagocytosis, etc. The proposed scheme for diagnosis of HPS recommends presence of at least five out of following nine criteria [13]:

- fever: peak temperature >38.5 °C for 7 or more days
- splenomegaly: spleen palpated >3 cm below the left costal margin
- cytopenia involving two or more cell lines: haemoglobin <9.0 g/dL, or platelet <100,000/µL, or absolute neutrophil count <1000/µL
- hypertryglyceridaemia or hypofibrinogenaemia: fasting triglycerides >2.0 mmol/L, or more than 3 standard deviations (SD) above the normal value for age, or fibrinogen <1.5 g/L, or more than 3 SD below the normal value for age
- haemophagocytosis: demonstrated in bone marrow, spleen, or lymph node; no evidence for malignancy
- hepatitis
- low or absent natural killer cell activity
- serum ferritin level >500 µg/L (although >3000 µg/L is a more realistic cut off to exclude infections and
- soluble CD25 (sIL-2 receptor) >2400 U/mL (note age-related norms).

Our patient had seven out of these nine criteria. Our center does not have the capacity to test for natural killer cell activity and soluble CD25.

Haemophagocytosis can be demonstrated among biopsy samples from bone marrow, liver or spleen, and bone marrow is an excellent sample for exhibiting haemophagicytosis associated with TB [2–4, 13]. As TB-associated HPS is rare, patients have been treated by different approaches. Brastianos et al. [8] in a review of 36 such cases found that anti-TB therapy alone showed better survival than combination of anti-TB drugs and various immunomodulatory therapies. In such cases, delay in diagnosis was found to be the most important factor for poor outcome [8]. Our patient responded well to anti-TB treatment, prednisolone was administered only in initial periods.

It should be mentioned that being a high TB-burden country, in our clinical practice we encounter many cases of disseminated TB even in immunocomtetent individuals. Delay in establishing an aetiological diagnosis or haematogenous spread may be contributory in this particular case for such an advanced presentation.

## Conclusion

Although associated with multiple conditions, TB should always be considered as cause of HPS in countries like Bangladesh where TB is endemic. An early diagnosis and treatment with appropriate anti-TB drugs is life-saving.

## Abbreviations

HPS: haemophagocytic syndrome
HLH: haemophagocytic lymphangiohistiocytosis
TB: tuberculosis
ALT: alanine aminotransferase
WHO: World Health Organization
BMI: body mass index
AST: aspartate aminotransferase
γ-GT: gamma-glutamyl transferase
LDH: lactate dehydrogenase
ECG: electrocardiography
ECHO: echocardiography
ICT: immunochromatography
HBsAg: hepatitis B virus surface antigen
HCV: hepatitis C virus
VDRL: veneral disease research laboratory
TPHA: treponema pellidum haemaglutinine assay
HIV: human immunodeficiency virus
ANA: antinuclear antibody
RF: rheumatoid factor
CMV: cytomegalovirus
CT: computed tomographic
SD: standard deviation
